# Quantitation and Stability of Nicotine in Canadian Vaping Liquids

**DOI:** 10.3390/toxics11040378

**Published:** 2023-04-17

**Authors:** Ivana Kosarac, Guru P. Katuri, Cariton Kubwabo, Shabana Siddique, Trevor K. Mischki

**Affiliations:** 1 Tobacco Control Directorate, Health Canada, Ottawa, ON K1A 0K9, Canada; 2 Environmental Health Science and Research Bureau, Health Canada, Ottawa, ON K1A 0K9, Canada

**Keywords:** e-liquids, e-cigarettes, nicotine, GC-MS, smoking, tobacco, matrix effects, vaping liquids, vaping products, stability study

## Abstract

Electronic cigarettes (e-cigarettes, vaping products) have become increasingly popular, with recent increases in use associated with closed systems delivering higher concentrations of nicotine. Most vaping products designed as an alternative to combustible cigarettes contain nicotine. A number of published studies have examined the reported concentrations of nicotine in vaping liquids (e-liquids) and found discrepancies between labelled and measured levels. Some discrepancy can also be explained by the lack of stability of nicotine in these types of products. Recently, a chemical analysis method for the quantitative determination of low and high levels of nicotine in vaping liquids was developed. This method uses dilution with acetonitrile prior to analysis with gas chromatograph mass spectrometry (GC-MS) in single ion monitoring mode (SIM). The developed method was validated using a laboratory-prepared vaping liquid as well as commercially available, nicotine-free products fortified with nicotine in the laboratory. The method detection limit (MDL) and the limit of quantitation (LOQ) for nicotine were calculated to be 0.002 mg/mL and 0.006 mg/mL, respectively. The newly developed method was applied to quantify nicotine in commercially available vaping liquids of various flavour profiles and across a wide range of nicotine concentrations, including those with nicotine salts. Furthermore, a subset of vaping liquids were analyzed to elucidate nicotine stability in various product subtypes. After a period of six months of accelerated storage to mimic one year, the overall mean percent of the original nicotine concentration remaining in the salt-based vaping products was 85% (minimum 64%, maximum 99%) while in the free-base nicotine products it was 74% (minimum 31%, maximum 106%). Nicotine stability in vaping liquids was found to be influenced by the nicotine form (pH) of formulation and its chemical composition. Non-targeted, qualitative analysis of chemical composition of vaping products showed that most constituents were identified and found to be remaining in the products following stability trials; however, three new compounds were tentatively identified in some vaping liquids at the end of the stability trials. Stability studies and the accurate quantitation of nicotine in vaping products can help inform product standards related to the safety, quality and utility of vaping products as a smoking cessation tool.

## 1. Introduction

The increased popularity of vaping products is evident. In 2020, 5.2 million Canadians aged 15 years and older reported having vaped [1]. Over the years, interest in the use of vaping products among youths aged 15 to 19 years old, in particular, has increased significantly, with 35% reporting to have ever tried vaping in 2020 compared to 23% in 2017 [2]. Over this same time period, the percentage of those aged 20 to 24 who have ever used vaping products also increased from 29% to 43%. This increased prevalence of use was not observed among those aged 25 years and older, with the rate holding around 13% in both 2017 and 2020. However, there was an increase in past 30 day use in this age group, from 2% in 2017 to 3% in 2020. Although this percentage increase may not appear as high, it in fact translates into around 264,000 more Canadians, or a total number of around 854,000 individuals aged 25 years and older who vape on a more regular basis. 

Nicotine-containing vaping products are battery-operated devices which heat and aerosolize a vaping liquid (e-liquid, Electronic Nicotine Device (ENDS) liquid). The liquids contain a mixture of carrier solvents, typically propylene glycol and vegetable glycerine, and in most cases flavouring chemicals and nicotine. Volatilized vaping liquid containing nicotine and flavours condenses an aerosol of liquid droplets that is inhaled by users. The health outcomes of exposure to flavours and solvents via inhalation have not been well studied to date, whereas the effects of nicotine and other toxicant exposures from tobacco products are well known [3]. The comprehensive report from 2018 on e-cigarette health effects by the National Academies of Science, Engineering and Medicine concluded that the use of vaping products under typical conditions results in lower levels of exposure to potentially toxic chemicals when compared to conventional tobacco cigarettes [4]. The same report also highlighted that nicotine exposure resulting from vaping can be very variable, highly dependent on the experience of the user and in some instances equivalent to conventional cigarette use.

Cases of accidental or intentional nicotine poisonings through the ingestion of vaping liquids have been recorded in the published literature [5,6,7]. Although there are limitations and uncertainties associated with determining the threshold for acute toxic effects, the estimated minimum lethal oral dose of nicotine is 0.8–1.0 mg/kg for adults [8]. The addiction potential and the exact amounts of nicotine required to create dependency are not well established due to ethical concerns in conducting such research; however, studies involving youths who smoke point to adolescents becoming dependent on nicotine with lower levels of exposure than adults [9]. The accurate labelling of nicotine concentration in vaping products is important to ensure that users are not misled and misinformed.

Vaping products are often chemically complex and sold in a variety of flavours (e.g., fruit, mint, tobacco, alcohol, etc.). In addition, a number of liquids are available as nicotine-free, i.e., those that specify 0 mg/mL nicotine concentration or declare explicitly not to contain nicotine, nor list the compound in the ingredients found on the product label or packaging material. In Canada, the maximum amount of nicotine allowed in the vaping liquids is 20 mg/mL. This limit was adopted in June of 2021 as a result of concerns regarding the availability of high-nicotine products and the role they may have played in contributing to a rapid rise in vaping among the youth [10]. Although vaping liquids were allowed to contain up to 66 mg/mL of nicotine prior to the concentration limit, until 2018, the majority of products were in fact below 18 mg/mL. Since 2018, new product types with higher concentrations of nicotine salts entered the market, and they achieved widespread popularity and larger market share [10]. Thus, the vaping product market in Canada is complex, variable and has changed significantly over a relatively short period of time. 

A number of studies measuring nicotine in vaping liquids have found that the actual nicotine concentration often differs substantially from the concentration labelled on the product [11,12]. Particularly concerning are instances where nicotine is found in vaping liquids labelled as nicotine-free due to the known risks of nicotine exposure [13,14]. The discrepancies between measured and labelled values have been attributed to improper manufacturing conditions and lack of standardization [15,16]. This discrepancy can also be explained by the lack of stability of nicotine in these product types. A few studies have reported on the stability of nicotine and its degradation products in vaping products to date [17,18,19]; however, the approaches were limited to thermal degradation for short periods of time, up to 2 h at 60 °C [18] and for 10 days at 60 °C [17].

Analysis using gas chromatography–mass spectrometry (GC-MS) provides an option to quantify nicotine without potentially interfering or co-eluting peaks. Given the variety of vaping products on the Canadian market, and particularly the wide range of nicotine concentrations, there is a need for a versatile method to measure nicotine at very low levels (ranging from nicotine-free to 1.5 mg/mL) as well as medium–high levels (2–70mg/mL) in samples with a high degree of matrix complexity. We aimed to address this need by developing a novel and versatile GC-MS method.

The developed GC-MS method was applied in a series of trials over a six-month period in order to better understand nicotine stability in vaping liquids. In addition, using the non-targeted chemical analysis, we monitored the overall chemical composition of select products to observe changes and chemical transformations during this time period.

## 2. Experimental Section

### 2.1. Chemicals and Reagents

Nicotine (≥99%) standard was purchased from Sigma-Aldrich (Oakville, ON, Canada). (±)-Nicotine-d_7_ (N-methyl-d_3_; pyridine-d_4_) was obtained from Canadian Isotopes (Pointe-Claire, QC, Canada). DL-Nicotine-^13^C_3_ (99%) was obtained from Cambridge isotope laboratories (Tewksbury, MA, USA), and 99.7% pure propylene glycol (PG) and 99.2% pure glycerol (VG) were purchased from Sigma-Aldrich (Oakville, ON, Canada). EMD Chemicals, Inc.’s acetone, methanol and acetonitrile of HPLC grade were purchased from Fisher Scientific (Ottawa, ON, Canada).

### 2.2. Samples

Vaping liquid samples were purchased between 2017 and 2019 in Canada. Although nicotine was quantified in 40 products, an additional subset of 11 samples were selected to further perform stability studies and monitor the chemical composition over time.

#### 2.2.1. Quantification Study

Forty vaping liquids were obtained through online and brick-and-mortar, commercial sources in Canada from various manufacturers. A majority of the liquids were 30 mL in size and packaged in child-resistant containers. The samples analyzed were split into two groups according to the labelled nicotine on the product: nicotine-free or low-nicotine group (<0.002–1.5 mg/mL, N = 12) and medium–high-nicotine group (2–70 mg/mL, N = 28). Seven of twelve nicotine-free or low-nicotine group vaping liquids were packaged in glass bottles, while others were packaged in plastic. All samples in this group, with the exception of two samples (#0084 and #0086), were labelled as nicotine-free or containing 0 mg/mL of nicotine. Samples #0084 and #0086 were labelled to contain 1.5 mg/mL nicotine.

In total, 28 vaping liquids were collected in the medium–high-nicotine group, ranging in labelled concentrations from 3 to 59 mg/mL. According to the product labels, ten vaping liquids in this category were in the form of nicotine salts; of these, five were packaged in a plastic pod style design, while all others were packaged in 30 mL child-resistant glass bottles.

#### 2.2.2. Stability Study

Two sets of vaping liquids, one set fortified with ^13^C_3_ and other one without isotope fortification, were chosen from various flavour profiles containing two different forms of nicotine, free-base (N = 6) and salt (N = 5). Vaping products of nicotine salt were of higher concentrations of nicotine (35–59 mg/mL), while free-base nicotine liquids were low to medium concentrations (6–18 mg/mL). Sample information such as flavour, nicotine form and concentrations, and product type are provided in Table 5.

### 2.3. Sample Preparation

#### 2.3.1. Quantification of Nicotine in Vaping Liquids

A total of ten milligrams of each vaping liquid were weighted to the nearest 0.1 mg. Each sample from a product labelled ≥1.5 mg/mL was fortified with 20 µL of 100 ng/µL (±) nicotine d_7_ internal standard solution (IS) in acetonitrile, followed by dilution with acetonitrile to 20 Ml and vortex mixing.

Samples from products labelled ≥1.5 mg/mL were first diluted to 20 mL with acetonitrile. Then, a 30 µL aliquot of each sample dilution was transferred to an autosampler vial and fortified with 10 µL of 10 ng/µL (±) nicotine d_7_ internal standard and made up to the volume of 1 mL with acetonitrile subsequently. One microliter of diluted sample was analyzed on the GC-MS.

Two sets of matrix-matched calibration standards, low (0.01–1.5 mg/mL) and medium–high (2–70 mg/mL), were prepared using the PG/VG (50/50 *w*/*w*), fortified with the corresponding volume of deuterated and native nicotine and diluted in acetonitrile in order to mimic the dilution and sample preparation of real samples.

#### 2.3.2. Stability Study

The stability study of nicotine-containing vaping liquids was performed according to “The International Council for Harmonisation of Technical Requirements for Pharmaceuticals for Human Use (ICH) guidelines on stability testing of new drug substances and products” [20]. Three different batches of each sample were stored in glass screw-top amber vials under thermolytic conditions (at 40 °C ± 2 °C, in the dark, under not more than 25% relative humidity) until analysis. The triplicates of each sample were analyzed at six different time intervals over 24 weeks to simulate the stability of nicotine under accelerated storage conditions and increase the rate of chemical degradation. This study was not focused on the stability of nicotine under photolytic, hydrolytic (acid/base) and oxidative stress conditions. Samples were withdrawn at predetermined intervals and subjected to sample preparation before analysis by GC-MS.

The above sample preparation method developed for the quantification study was also applied to the stability study with slight modifications. Briefly, 0.17 g of vaping liquid was mixed with 0.03 g of 50 mg/mL (+) nicotine ^13^C_3_ solution in 50/50 (*w*/*w*) propylene glycol/vegetable glycerine (PG/VG) followed by vortex mixing. At time points 0, 1, 6, 12, 18 and 24 weeks, 10 mg aliquots were taken from samples and further diluted with 20 mL of acetone. A total of 30 µL of diluted vaping liquid was added to 10 µL of 10,000 pg/µL d_7_ nicotine internal standard and transferred to 960 uL of acetone, followed by vortex mixing. Some of the samples were processed without adding the nicotine ^13^C_3_ in order to monitor if, through fortification with isotope-labelled nicotine, overall chemistries would be unintentionally affected.

##### Stability Study—Qualitative, Non-Targeted Analysis

A total of 40 microliters of each study sample, before and after stability trials, was collected and diluted to 20 mL with methanol. Diluted aliquots were mixed thoroughly using the vortex mixer, and 1 µL was injected and analyzed using GC-MS. Methanol solvent blank was injected after each sample to ensure there was no carryover between samples. In addition, matrix blank consisting of propylene glycol and glycerol was used to assess possibility of PG/VG thermal degradation during GC analysis.

### 2.4. GC-MS Analysis

#### 2.4.1. Quantitative, Targeted Analysis

The chromatographic separation was performed on a Zebron ZB-5MS capillary column (30 m × 0.25 mm × 0.25 µm) from Phenomenex (CA, USA), using an Agilent 7890 GC gas chromatograph equipped with an Agilent 7693B Series Autosampler. A 1 µL sample was injected in splitless mode at 300 °C. The GC oven temperature program was as follows: 100 °C to 200 °C at 10 °C/min, followed by 15 °C/min to 310 °C and holding for 3 min. Helium was the carrier gas at a flow rate of 1 mL/min in constant flow mode. Mass spectrometry analysis was performed using a Waters Quattro micro-GC triple quadrupole mass spectrometer (Waters Corp., Milford, MA, USA). The source and GC interface temperatures were set at 180 and 250 °C, respectively. The MS was operated in electron ionization at 70 eV in single ion recording (SIR) mode. MassLynx version 4 was used for data acquisition and processing. The following single ions were monitored: nicotine (*m*/*z*) 84 and 162, quantifier and qualifier ions, respectively, nicotine d7 (*m*/*z*) 87 and 169, quantifier and qualifier ions, respectively, and nicotine 13C3 (*m*/*z*) 165 and 134, quantifier and qualifier ions, respectively.

#### 2.4.2. Qualitative, Non-Targeted Analysis

The procedure for non-targeted analysis has been previously described [21]. In summary, samples were characterized using the gas chromatogram tandem mass spectrometer operated in a full scan mode. Each spectrum obtained was matched against mass spectral libraries (National Institute of Standards and Technology (NIST 17) and Wiley’s library of Mass Spectra of Flavors and Fragrances of Natural and Synthetic Compounds (FFNSC)), while for some compounds, direct matching against genuine analytical standards was used. An internal analytical standards library of expected compounds was created based on previous reports from the published literature on the occurrence of various analytes in the vaping liquids.

## 3. Results and Discussion

### 3.1. Matrix Effects

In the case of vaping liquids, nicotine is present in a matrix most commonly consisting of propylene glycol and vegetable glycerin. GC-MS is considered to be a powerful analytical technique to quantify nicotine in tobacco products. However, this technique, when used to quantify low levels of analyte in complex mixtures, may result in signal suppression, enhancement and interference due to matrix effects. In the initial stage of method development, strong matrix interferences were observed with respect to absolute nicotine responses (Figure S1).

The relatively low amount of PG and VG (0.05% *v*/*v*) in the pure standard of nicotine dissolved in acetonitrile enhanced the absolute nicotine signal significantly when compared to the pure standard of the same nicotine concentration dissolved in acetonitrile without PG and VG added. To overcome the problem of matrix effects, we chose the matrix-matched calibration and also included the isotopically labelled internal standard (nicotine d_7_) to measure low levels of nicotine in vaping liquids very accurately.

### 3.2. Method Performance and Validation

The method detection limit was assessed according to the EPA Regulation 40 CFR part 136 (Appendix B) method [22]. Eight replicates of laboratory-prepared vaping liquids using USP-grade PG and VG were fortified to a low nicotine concentration of 0.05 mg/mL, and then diluted with acetonitrile and analyzed. The standard deviation associated with eight replicate analyses of laboratory-prepared vaping liquid and processed through the entire analytical procedure was multiplied by the Student’s *t* value of 2.998 (appropriate for a 99% confidence level with 7 degrees of freedom). The method detection limit (MDL) for nicotine was calculated to be 0.002 mg/mL. The limit of quantitation (LOQ) was calculated according to the US EPA method, where the standard deviation associated with the eight replicate analyses of laboratory-prepared vaping liquids conducted to obtain the MDL was multiplied by a factor of 10. The LOQ was calculated to be 0.006 mg/mL. The relative standard deviation for eight repetitions of this laboratory-prepared vaping sample was 3.14.

Laboratory blanks consisting of acetonitrile as well as laboratory-prepared nicotine-free vaping liquid containing PG/VG (50/50 *v*/*v*) were used to assess between injection carry-over and possible nicotine contamination.

Three commercially available vaping liquids which were analyzed and confirmed not to have any detectable nicotine levels were spiked at nicotine concentrations of 25 mg/mL to further investigate the matrix effects and applicability of the method to the higher-concentration vaping liquids. The vaping liquids were of various PG/VG proportions and triplicate analysis of each yielded satisfactory results with respect to repeatability and accuracy when nicotine was present at higher concentrations (Table 1).

Each sample batch analyzed on a given day included two measurements of a quality control (QC) sample, i.e., a laboratory-prepared vaping liquid consisting of 50/50 PG/VG and containing nicotine at 20 mg/mL.

The calibration curve using nicotine d_7_ as an internal standard was linear over the range of 0.01–1.5 mg/mL (representing 5–750 ng/mL diluted concentration) for nicotine with r^2^ = 0.9998. For vaping liquids in medium–high-nicotine categories, the calibration curve using nicotine d_7_ as an internal standard was linear over the range of 2–70 mg/mL (representing 30–1050 ng/mL diluted concentration) of nicotine with r^2^ = 0.9993. Average recoveries were 82%.

### 3.3. Method Application

#### 3.3.1. Nicotine-Free or Low-Nicotine Vaping Liquids Analysis

The newly developed method was first applied to twelve vaping liquids purchased on the Canadian market from online and brick-and-mortar stores. Ten of the twelve vaping liquids were labelled as nicotine free and two were labelled as containing nicotine at 1.5 mg/mL (Table 2).

**Figure 1:**
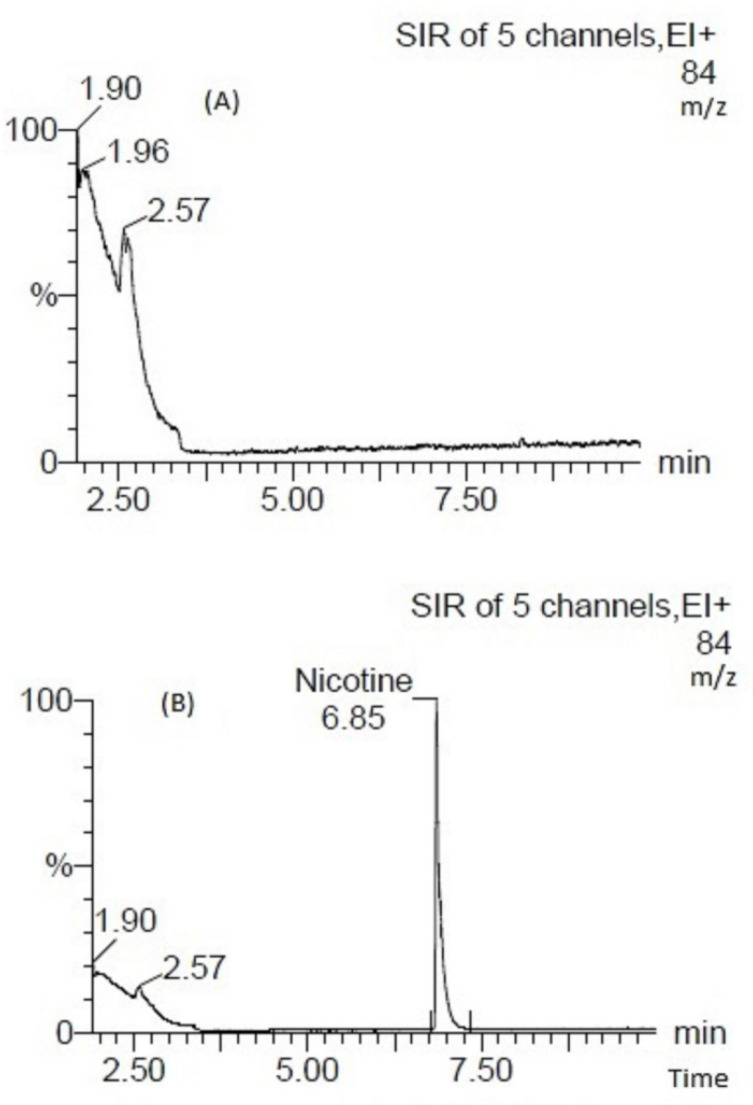
Vaping liquid extracted by mass chromatogram for single ion mass monitoring, *m*/*z* 84 in (**A**) sample 0061 nicotine-free vaping liquid (floral flavour category), (**B**) sample 0084 labelled as 1.5 mg/mL nicotine vaping liquid (confectionery category).

Eight vaping liquids labelled as nicotine-free did not have any detectable levels of nicotine (Figure 1A); however, two vaping liquids were found to contain nicotine at concentrations of 0.009 and 0.008 mg/mL, both of which are above the MDL and LOQ. Two vaping liquids (0084 and 0086) labelled as 1.5 mg/mL were measured to contain nicotine at 0.967 mg/mL and 0.968 mg/mL, respectively (Figure 1B). These two products were manufactured by the same manufacturer, obtained from an online store and purchased before the enactment of the Tobacco and Vaping Products Act (Government of Canada 2018), suggesting that samples, perhaps, were not of a high quality, as there was no legislation to govern product manufacturing at that time.

#### 3.3.2. Medium- and High-Nicotine Vaping Liquids Analysis

The extended nicotine concentration range analysis was applied to 28 samples in the medium–high-nicotine category. Two vaping liquids were measured to contain nicotine only slightly above the nicotine level labelled. Measured nicotine concentrations for twenty-six (93%) vaping liquids in this category were found to contain lower concentrations than what was labelled on the products, and the nicotine concentration of five vaping liquids was within a 20% difference in the labelled nicotine concentrations (Table 3). Similarly, in another study [14] on Canadian vaping products, Czoli et al. also found lower measured nicotine concentrations compared to the product labels in 85% of samples analyzed. In fact, lower-than-labelled nicotine is frequently observed in most published studies (Table S1), with the exception of a study [17] from New Zealand where all nicotine-containing products (N = 10) were found to contain higher levels than labelled.

The reason for lower measured nicotine concentrations could be the nicotine degradation during the ageing process of vaping liquids or, alternatively, a result of poor manufacturing processes. Hence, conducting the stability trials of nicotine in vaping liquids is very important in order to better understand these discrepancies between measured and labelled concentrations.

It is worth noting that the nicotine salt vaping liquids were not studied in great detail to understand the proportion of nicotine present as a free base (Nic) and as protonated forms (NicH^+^); in fact, assumptions were made that the protonated forms were, as free-base, soluble in acetonitrile and methanol, as our methodology is mainly dilution- and not extraction-based. The pH of individual vaping liquids was not measured and not used to estimate ratios of different forms of nicotine in the vaping liquids, as according to a study by El-Hellani et al. [23], the correlation between pH measurements and actual vaping liquid experimental measurements of the ratio of free-base and protonated forms of nicotine is very weak and highly dependent on the presence of other constituents in the matrix. When we compared the deviances in the difference between measured concentrations and labelled concentrations of the two groups of vaping liquids (free-base-nicotine-containing liquids vs. nicotine-salt-containing liquids) using the Brown–Forsythe test of homogeneity of variances (modified Levene’s test), the differences were not statistically significant (*p*-value = 0.051).

Table 4 provides a summary of published methods along with ranges of measured nicotine in the commercially available vaping liquids analyzed in each study. Published studies that measured low-nicotine or nicotine-free vaping liquids are marked with “nicotine-free” in parentheses under the range heading. With respect to limits of detection, dynamic range and simplicity of sample extraction, the newly developed method compares well with other published methods on nicotine quantification in vaping liquids.

#### 3.3.3. Nicotine Stability Study

The quantification method developed using GC-MS was successfully applied for stability trials to quantify nicotine and separate it from degradation products in the vaping liquids studied. The method is rapid, simple, inexpensive and does not require the use of hazardous chemicals.

Nicotine-containing vaping liquids also contain nicotine-related minor alkaloids. In our stability studies, these compounds were observed to be a source of nicotine upon the heating of the vaping liquid. In order to unequivocally measure the nicotine stability, samples were fortified with known concentrations of ^13^C_3_-labelled nicotine to ensure that no other alkaloids or nicotine oxides present in these products would contribute to the overall concentrations measured. Therefore, both native (^12^C) nicotine and the unnatural form, ^13^C_3_ nicotine, were measured and compared. Most vaping liquid samples were stable for 1 week at 40 °C and started degrading afterwards. In all samples assayed, after degradation over a period of 6 months, the amount of nicotine present (mg/mL) was lower than the labelled content except for two products (Mint salt and Mint free-base, Table 5).

The reasons for lack of degradation in the two mint-flavoured products warrants a closer look and further examination in the future, especially since they contained two different forms of nicotine, and in both cases, ^13^C_3_ nicotine was degrading at a similar rate to that observed in the other flavoured products studied. The presence of other ingredients in products may have a stabilizing effect on the formulations; for example, mint extract is well known to contain phenolic and other compounds with antioxidant and antimicrobial properties [35,36]. Non-targeted analysis of mint-flavoured vaping products confirms the presence of compounds such as menthol, carvone and cis-ocimenol (Table S2), which may play a role in reduced degradation. Conversely, aqueous solutions may induce hydrolysis during the thermal degradation process. Water is often added to the vaping formulations, and given that propylene glycol and glycerol, major vaping liquid components, are hygroscopic, the introduction of water may make products more prone to the degradation of nicotine.

**Figure 2:**
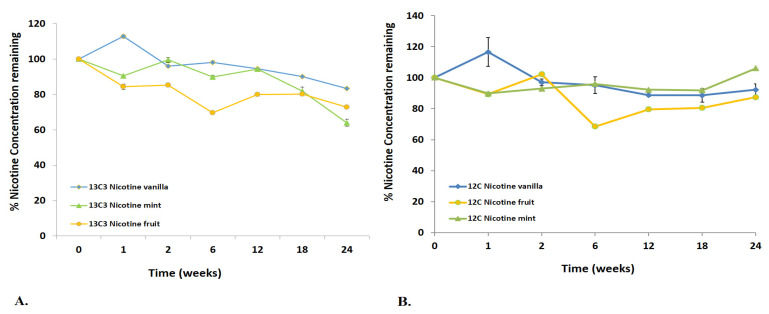
(**A**)**.** Stability of 13C_3_ nicotine; (**B**). Stability of ^12^C nicotine in three lots each: vanilla, mint and fruit vaping products.

In simple laboratory-prepared vaping liquids containing only PG/VG 50/50 (*w*/*w*) and nicotine, nicotine concentration degraded to 76% remaining over a 6-month stability study period. For the majority of products studied, ^13^C_3_ nicotine degraded at a higher rate than native nicotine in the vaping products over this period (Figure 2).

The mean percent of the original nicotine concentration remaining, overall, in salt-based vaping products was 85%, and in free-base nicotine products 74%. Although we tested a limited number of products, one possible explanation for more stable nicotine salt products is a lower pH and, presumably, the presence of organic acid, which renders the nicotine more stable. Similar observations were reported in stress studies of nicotine [17] where, under alkaline conditions, the active compound was more readily degraded: 88% after 5 days, compared with 97% remaining after 10 days under acidic conditions. Nicotine remaining after 6 months of simulated degradation in salt-based formulations ranged between 64% (tobacco-flavoured product) and 99% (mint-flavoured product), while among the free-base products remaining, concentrations ranged between 31% (tea-flavoured product) and 106% (mint-flavoured product).

**Figure 3:**
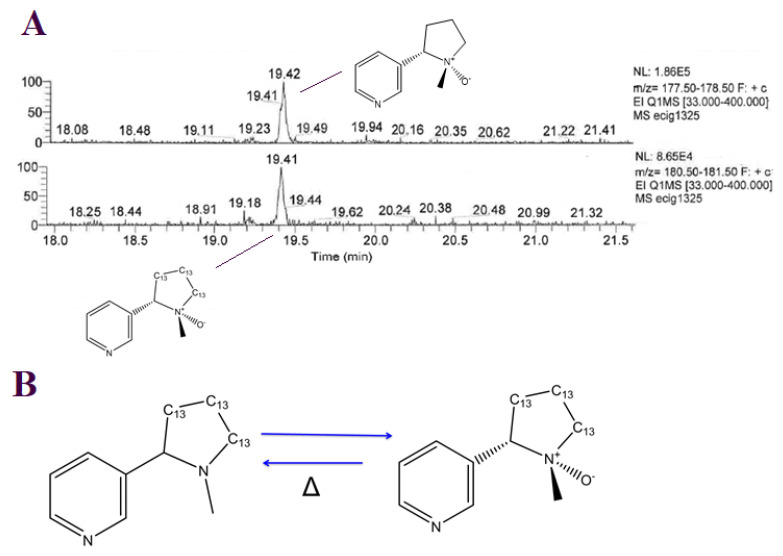
(**A**)**.** Extracted ion chromatograms of (1′S 2′S) nicotine-1′-oxide (m/z 178) and (1′S 2′S) 13C_3_ nicotine-1′-oxide (m/z 181); (**B**)**.** Degradation and thermal rearrangement of ^13^C_3_ nicotine to (1′S 2′S) ^13^C_3_ nicotine-1′-oxide.

In order to better characterize the transformation of chemical compounds and observe the presence of new compounds formed at the end of the stability studies, vaping liquids were also analyzed using the non-targeted mass spectrometry method (Figure S2). In the majority of cases, (1′S 2′S) nicotine-1′-oxide appeared to be the major degradation product. This observation was proven, as we were able to detect (1′S 2′S) ^13^C_3_ nicotine-1′-oxide generated during the degradation of the unnatural and fortified ^13^C_3_ isotope of nicotine in samples which underwent the nicotine degradation (Figure 3A). Nicotine oxides, in turn, are thermally labile and will, upon heating, presumably reduce back to nicotine at temperature-dependent rates [37] (Figure 3B).

Although we were able to detect the oxide form, we did not quantify it, as gas chromatography methods are not optimal for this purpose given the high temperatures used during the analysis. In future studies, liquid-chromatography-based techniques will be optimized to quantify the oxides as well. Another degradant, a nicotine oxidation byproduct and impurity, β-Nicotyrine, was also detected in 8 out of the 11 products studied. Unlike (1′S 2′S) nicotine-1′-oxide, β-Nicotyrine was detected in the liquids at the start of the stability studies, indicating that it may have originated from the tobacco plant nicotine extract or formed during product storage [38]. This presence of oxides and other alkaloids in samples prior to the initiation of the stability study may be why the free-base mint product’s native nicotine concentration was lower before the degradation period, with 106% remaining after degradation.

Non-targeted analysis (Table S2) revealed that the majority of chemical constituents of the liquids studied remained present in the formulations over six months. Apart from nicotine, none of the other analytes were quantified to determine the relative concentration changes over the stability study period; therefore, no conclusions can be made with respect to changes in the concentrations of other chemicals present in the products. Of note is that three new compounds were detected and tentatively identified in some products at the end of the degradation study: Cinnamaldehyde Propylene Glycol Acetal (one product), Diglycolic acid, ethyl 2-isopropylphenyl ester (four products) and butanedioic acid, 2,3-dimethoxy-, diethyl ester (three products). While the origin and formation of the two esters of carboxylic acids are unknown, cinnamaldehyde PG acetal formation has been reported to form in vaping liquids elsewhere [39,40].

In the previously reported degradation studies, experiments were performed at different temperatures and for various time periods [17,18,19], since the ICH and USP guidelines for stability testing do not specify the limits of degradation in the forced degradation studies. For instance, in a study by Bansal et. al., the experiment was performed at 60 °C for 10 days. At this temperature, the aqueous nicotine solution showed slow but marked degradation after 10 days, while most vaping liquids remained stable, with less than 5% degradation over the same time period. In a study by Gholap et al., thermal degradation was carried out at 60 °C for 2 h, and more than 5% degradation was observed in each of the vaping liquids studied. In stability trials by Kim et al., nicotine in vaping liquids was assessed by the color stability at different temperatures for a shorter period of time. Under the thermocycling conditions (21 C° for 90 s and 50 C° for 90 s over 24 h and 72 h time periods), subsamples remained homogenous up to 72 h without secondary separation compared to a control; at day 7, the color of the vaping liquid began to change from clear to yellow. In general, a degradation of 5–20% of active compound in at least one of the stressed conditions is the acceptable range of forced degradation [41]. In our study, 4 (1 salt and 3 free-base nicotine) out of the 11 products were found to have degraded over 20% in a six-month period.

One of the limitations of this study includes the lack of information on the duration of and conditions in which analyzed samples were stored prior to purchase, while still at the manufacturers’ facilities or vape shops. Only four (001, 0193, 0219, 0220) products out of the fifty-one studied had clearly marked manufacturing dates that could be used to estimate the duration these products spent “on shelf” at the manufacturers’ premises prior to sample collection. All four samples were refillable products packaged in 30 mL transparent glass bottles with no overpackaging, likely exposed to light. One sample (001) was manufactured seven months prior to purchase, and three samples (0193, 0219, 0220) were manufactured four months prior to sample purchase. In order to account for a possible degradation during the time between manufacturing and purchasing dates, as explained earlier, nicotine was measured at the beginning of the study to confirm the actual concentration; additionally, isotopically labelled nicotine was fortified to monitor the degradation process. Another limitation of the study is the lack of knowledge around the duration of the time users take to consume the product once it is opened. Although low-volume, pod-based products are presumably consumed in a short period of time, it would be important to know for how long large-volume (e.g., 60 or 100 mL) refillable formats could be used. Of note is that the users may purchase a variety of flavoured products and not consume from a single bottle daily but instead prolong the opened bottle’s consumption time by switching flavours used in their device on different days to avoid reduction in sensation of flavours reported among people who vape elsewhere (“vaper’s tongue or fatigue”) [42,43]. Therefore, it is unclear whether the time between product manufacturing and complete consumption by the user, in real life, is well represented by an accelerated, general storage, degradation study conducted here over a six-month period. Finally, this study did not analyze the aerosol derived from the vaping liquids examined. Complexities introduced through aerosolization and the widely variable puffing parameters of the vapers should be recognized as a source of varied exposure compared to chemicals identified in vaping liquids.

Understanding the concentrations of nicotine present in various products is important to estimate nicotine yield in emissions and the ultimate amount available for users to inhale. Lower-than-labelled concentrations significantly degraded nicotine, such as the example of the tea-flavoured product analyzed in our study (69% degraded), which may have negative implications on the use of vaping products as potential cessation tools in that they may not provide nicotine satiation and discourage people looking to quit smoking using vaping. Of note is that we were able to find labelled product expiry dates for only 43% (22 out of 51) of the products studied, indicating a lack of this important product quality information that should be communicated to the consumers.

## 4. Conclusions

A sensitive and accurate method for the quantification of nicotine in vaping products was developed by GC–MS. The main challenge in developing the method is the lack of certified reference material for vaping liquids. The number of vaping liquid flavours currently available in Canada is vast. It is, in fact, this product heterogeneity that prevents the chemical analysis of one vaping liquid to be broadly applicable to others. In the absence of commercially available reference material, laboratories should prepare their own to best simulate the matrix of vaping liquids that will be analyzed. Through the use of the matrix-matched calibration, this method has demonstrated precision with detection limits of 0.002 mg/mL and linearity with a wide dynamic range from 0.01 to 70 mg/mL, respectively. The measured concentrations of vaping liquids quantified were generally lower than the labelled concentrations. The reason for lower measured nicotine concentrations could be due to the result of poor manufacturing processes as well as nicotine degradation during the ageing process of products, as demonstrated through the stability trials of nicotine in vaping liquids under accelerated storage conditions at 40 °C as per ICH guidelines.

After six months of degradation, the mean percent of the original nicotine concentration was 15% and 26% for salt- and free-base nicotine products, respectively. In some products studied, ^13^C_3_ nicotine degraded at a higher rate than native nicotine, suggesting, perhaps, that for some product types there may be other chemical constituents, such as nicotine-related alkaloids or oxides, that through chemical transformations may influence the overall amount of nicotine present in the product at any given time point. Future activities and efforts should be directed at better understanding product quality and the role of various chemical compounds, such as detected alkaloids, play for smoking cessation efficacy, addiction and safety of these products. Stability study data on the transformation and degradation of nicotine, other alkaloids and flavours in vaping products can be used to better inform standards, regulation and users looking to quit smoking using vaping.

## Figures and Tables

**Table 1:** Nicotine-free vaping liquids fortified at 25 mg/mL.

Consumable ID	Flavour	Nicotine Strength (mg/mL)	PG/VG Proportion	Packaging	Bottle Size (mL)	Fortified Nicotine Concentration in Vaping Liquid (mg/mL)	Measured Nicotine Concentration (mg/mL) (N = 3)	RSD	% Error
0005	Fruit	0	50/50	glass	30	25	23.1	1.7	7.6
0040	Fruit	0	30/70	glass	60	25	22.83	3.3	8.7
0056	Coffee	0	60/40	glass	30	25	22.19	1.7	11.2

RSD, relative standard deviation.

**Table 2:** Nicotine-free or low-nicotine vaping liquids analyzed, (N = 12).

Sample ID	Flavour Category	Nicotine Form	Product Type	PG/VG Content	Labelled Nicotine Concentration (mg/mL)	Measured Nicotine Concentration (mg/mL)
0001	Flavourless	n.a.	Refillable	20/80	0	<0.002
0002	Flavourless	n.a.	Refillable	50/50	0	<0.002
0003	Flavourless	n.a.	Refillable	70/30	0	<0.002
0005	Fruit	n.a.	Refillable	50/50	0	<0.002
0010	Tobacco	n.a.	Refillable	50/50	0	0.009
0023	Floral/herbal	n.a.	Refillable	20/80	0	0.008
0040	Fruit	n.a.	Refillable	30/70	0	<0.002
0052	Other	n.a.	Refillable	40/60	0	<0.002
0056	Coffee	n.a.	Refillable	60/40	0	<0.002
0061	Floral/herbal	n.a.	Refillable	>75% VG	0	<0.002
0084	Confectionary	Free-base	Refillable	50/50	1.5	0.967
0086	Tobacco	Free-base	Refillable	50/50	1.5	0.968

n.a.—Not applicable; although samples 0010 and 0023 had detectable levels of nicotine, it is unclear what form the nicotine is in.

**Table 3:** Medium- and high-nicotine vaping liquids analyzed, (N = 28).

Sample ID	Flavour	Nicotine Form	Product Type	PG/VG	Labelled Nicotine Concentration (mg/mL)	Measured Nicotine Concentration (mg/mL)
0006	Dessert	Free-base	Refillable	70/30	9	8.81
0007	Dessert	Free-base	Refillable	70/30	24	21.88
0008	Confectionary	Free-base	Refillable	50/50	12	10.59
0009	Confectionary	Free-base	Refillable	30/70	3	2.30 *
0011	Tobacco	Free-base	Refillable	30/70	15	15.09
0012	Mint/Menthol	Free-base	Refillable	70/30	24	17.30 *
0013	Mint/Menthol	Free-base	Refillable	100 VG	9	1.75 *
0015	Alcohol	Free-base	Refillable	70/30	24	12.80 *
0016	Soft drink	Free-base	Refillable	70/30	6	6.02
0017	Soft drink	Free-base	Refillable	Unknown	12	9.15 *
0018	Coffee	Free-base	Refillable	50/50	24	14.76 *
0019	Coffee	Free-base	Refillable	70/30	3	2.17 *
0020	Tea	Free-base	Refillable	70/30	9	6.80 *
0021	Tea	Free-base	Refillable	30/70	3	1.57 *
0022	Floral/Herbal	Free-base	Refillable	75 VG–97 VG	12	6.51 *
0024	Energy drink	Free-base	Refillable	70/30	6	4.77 *
0025	Energy drink	Free-base	Refillable	70/30	18	11.39 *
0026	Cereal	Free-base	Refillable	100 VG	24	19.43
0049	Mint/Menthol	Salt	Refillable	50/50	40	28.02 *
0083	Dessert	Salt	Refillable	50/50	20	15.54 *
0193	Fruit	Salt	Refillable	50/50	35	28.43
0219	Tobacco	Salt	Refillable	50/50	35	26.56 *
0220	Mint/Menthol	Salt	Refillable	50/50	35	27.23*
Pod cartridge 1	Mint/Menthol	Salt	Pod	Unknown	59	45.40 *
Pod cartridge 2	Fruit	Salt	Pod	Unknown	59	42.46 *
Pod cartridge 3	Dessert	Salt	Pod	Unknown	59	39.39 *
Pod cartridge 4	Tobacco	Salt	Pod	Unknown	59	40.50 *
Pod cartridge 5	Soft drink	Salt	Pod	50/50	12	6.43 *

* Results denote % differences in measured and labelled values higher than 20%.

**Table 4:** Summary of published methods for quantification of nicotine in vaping liquids.

Publication	LOD (mg/mL)	LOQ (mg/mL)	MDL (mg/mL)	Method of Extraction	Method Instrumentation	Range (mg/mL)
[24]			0.04	Dilution 1:100 (methanol)	GC-FID	8.65–13.1
[13]		0.05	0.05	Dilution: 1:100 (methanol)	GC-NPD	<LOQ-150.3
[25]	0.0002	0.0006	0.06	Dilution 1:100 (methanol)	GC-MS	
[23]				Liquid–liquid extraction with toluene and pH adjustments with ammonium hydroxide	GC-MS	0.05–1.5
[26]	1.49 × 10^−7^	4.52 × 10^−7^	0.00004	Dilution 1:400 with acetonitrile	GC-MS	
[27]	0.0001		0.0001	Extraction: alkalinized (sodium hydroxide)-->Liquid/liquid (dichloromethane/hexane 1:1)	GC-MS	0.0001–0.324 (nicotine-free)
[16]	0.05		0.05	Extraction: alkalinized (sodium hydroxide)--> Liquid/liquid (methyl tert-butyl ether)	GC MS/MS	<0.05–20.5
[28]		0.04		Solid Phase Extraction—no details provided	LC UV	0.19–24 (0.19–0.48 nicotine-free)
[12]	0.0001	0.00025		Dilution with 10% acetonitrile with 20 mM ammonium acetate or 10% acetonitrile for cig-alike devices; details not given for e-liquid refill solutions	LC UV	0.08–21.82
[29]	0.00001–0.00003			Dilution with 1 M ammonia to approximately 150 µg/mL	UHPLC UV PDA	ND-29
[30]		7.30 × 10^−5^	0.004	Dilution 1:50 with water for samples lower than 0.2 mg/mL; further dilution for samples > 0.2 mg/mL	LC MS	
[15]	3.00 × 10^−7^	1.00 × 10^−6^	0.001	Dilution 1:1000 (methanol)	LC MS/MS	<LOQ-0.254 (nicotine-free)
[31]		0.00001	0.2 to 0.5	Dilution 1:20,000 to 1:50,000 (1:9 water/methanol)	HPLC MS/MS	4.3–14.7
[32]	9.00 × 10^−6^			None	Ion mobility spectrometry (IMS)	<LOQ-26.4
[33]	0.0016	0.0055	0.055	Dilution: 1:10 (8:1 water/NMR buffer)	NMR	0.11–6.9 (nicotine-free)
[34]	1.62 × 10^−10^			Acidification with hydrochloric acid and dilution with methanol	FT ICR MS	
This study		0.006	0.002	Dilution 1:2000 with acetonitrile	GC MS/MS used in SIR to mimic GC-MS	<0.002–0.968

LOD: Limit of detection, LOQ: Limit of quantitation, MDL: Method detection limit, GC: Gas chromatography, LC: Liquid chromatography, MS: Mass spectrometry, UHPLC: Ultra-high liquid chromatography, FTICR: Fourier-transform ion cyclotron resonance, FID: Flame ionization detection, NPD: Nitrogen–phosphorus detectors, UV: Ultraviolet detector, PDA: Photo diode array, NMR: Nuclear magnetic resonance.

**Table 5:** Concentrations of nicotine and characteristics of products used in stability trials.

Product	Nicotine Form	Nicotine Conc. on Product Label (mg/mL)	Initial Measured Concentration (mg/mL)	RSD INITIAL Measured Conc.	Final Measured Concentration (mg/mL)	RSD Final Measured Conc.	% Remaining after 6 Months	Product Type	% Difference Label vs. Initial Measured Concentrations
Dessert vanilla	Salt	59	52.73	2.9	48.65	7.6	92.3	Pod	−11.89
Mint salt	Salt	59	52.43	2.8	51.98	9.2	99.1	Pod	−12.53
Tobacco 1	Salt	59	58.52	2.8	37.22	7.4	63.6	Pod	−0.82
Fruit mango	Salt	59	55.17	5.5	44.7	16.53	81	Pod	−6.94
Tobacco 2	Salt	35	36.4		33.5		92	Pod	3.85
Mint free-base	Free-base	18	14.71	9.4	15.59	23.3	106	Pod	−22.37
Fruit grape	Free-base	18	17.59	19.1	15.37	3.1	87.4	Refillable	−2.33
Tobacco 3	Free-base	18	20.04		11.22		56	Pod	10.18
Dessert fruit	Free-base	6	7.2	8.2	5.43	20.1	75.4	Refillable	16.67
Tea	Free-base	12	15.27		4.78		31.3	Refillable	21.41
Tobacco 4	Free-base	24	23.16		20.32		87.7	Refillable	−3.63

## Data Availability

The data presented in this study are available on request from the corresponding author. The data are not publicly available due to privacy.

## Supplementary Materials

The following supporting information can be downloaded at: https://www.mdpi.com/article/10.3390/toxics11040378/s1, Figure S1: Nicotine matrix effects (A) pure nicotine standard in acetonitrile (B) pure nicotine standard in acetonitrile with PG/VG present; Figure S2: Fruit sample (“Frozen Grape”) chromatogram generated through non targeted analysis prior to degradation; Table S1: Published studies showing % of products studied with lower nicotine concentration than declared on the vaping liquid label; Table S2: Chemical compounds detected through Non-targeted analysis in mint products before and after degradation
