# Supplementary material for: Quantitation and Stability of Nicotine in Canadian Vaping Liquids
Source: Toxics. 2023 Apr 17;11(4):378. doi: 10.3390/toxics11040378 (PMC10144332; doi:10.3390/toxics11040378)
Supplement: Supplementary file 1 [file toxics-11-00378-s001.zip › toxics-2257191-SI.pdf]

Supplementary Information

Quantitation and Stability of Nicotine in Canadian Vaping Liquids

Ivana Kosarac [1,*], Guru P. Katuri [1], Cariton Kubwabo [2], Shabana Siddique [2] and Trevor K. Mischki [1]

[1] Tobacco Control Directorate, Health Canada, Ottawa, ON K1A 0K9, Canada
[2] Environmental Health Science and Research Bureau, Health Canada, Ottawa, ON K1A 0K9, Canada
* Correspondence: ivana.kosarac@hc-sc.gc.ca

Table S1 Published studies showing % of products studied with lower nicotine concentration than declared on the vaping liquid label

Study	**Year**	**Total vaping liquids tested**	**% Products with measured nicotine lower than labelled**
(Czoli et al. 2018)	2018	73	85
(Bansal et al. 2018)	2018	12	0
(Famele et al. 2017)	2017	58	88
(Pagano et al. 2016)	2016	4	75
(Peace et al. 2016)	2016	27	74
(Crenshaw et al. 2016)	2016	18	78
(Goniewicz et al. 2015)	2015	91	81
(Kavvalakis et al. 2015)	2015	263	84
(Lisko et al. 2015)	2015	36	81
(Etter, Zäther, and Svensson 2013)	2013	40	65
This study		40	90

Table S2 Chemical compounds detected through Non-targeted analysis in mint products before and after degradation

Mint Salt nicotine product

Peak	Name	CAS	Detected before (B) and/or after (A) degradation	Notes - Flavour Descriptor
**1**	Propylene Glycol	57-55-6	B, A	Processing, carrier agent
**2**	Diglycolic acid, ethyl 2-isopropylphenyl ester		A	Data poor chemical
**2**	Benzyl alcohol	100-51-6	B, A	Additive, solvent, flavour
**3**	1, 8-Cineol	470-82-6	B, A	Flavour minty camphoreous cooling eucalyptus medicinal
**4**	Glycerin	56-81-5	B, A	Processing, carrier agent
**5**	cis-Pulegol	22472-80-6	B, A	Natural product found in Mentha arvensis and Mentha spicata
**6**	Menthone	89-80-5	B, A	Flavour cooling mentholic minty woody

**7**	l-Menthone	14073-97-3	B, A	Flavour cooling, peppermint, fresh green, minty with an herbal nuance
**8**	Menthol	15356-70-4	B, A	Flavour cooling mentholic minty
**9**	Benzoic acid	65-85-0	B, A	Organic acid
**10**	Neo-menthol	2216-52-6	B, A	Flavour cooling mentholic minty peppermint
**11**	cis-ocimenol	7643-60-9	B, A	Natural product
**12**	Carvone	99-49-0	B, A	Minty licorice odour
**13**	Piperitone	89-81-6	B, A	Flavour minty cooling mentholic spicy peppermint peppery
**14**	Menthyl acetate	16409-45-3	B, A	Tea cooling minty fruity berry odour
**15**	1,3-Diacetin	105-70-4	B, A	Flavour enhancer, processing agent
**16**	s-Nicotine	54-11-5	B, A	Nicotine
**17**	beta-Nicotyrine	487-19-4	B, A	Nicotine impurity
Free Base Nicotine Product				

Peak	Name	CAS	Detected before (B) and/or after (A) degradation	Notes - Flavour Descriptor
**1**	Propylene Glycol	57-55-6	B, A	Processing, carrier agent
**2**	Glycerin	56-81-5	B, A	Processing, carrier agent
**3**	Estragole	140-67-0	B, A	Flavour sweet, licorice, phenolic, weedy, spice, celery-like
**4**	l-Menthone	14073-97-3	B, A	Flavour cooling, peppermint, fresh green, minty with an herbal nuance
**5**	Neoisomenthol	490-99-3	B, A	Mentholic odour
**6**	l-Carvone	6485-40-1	B, A	Flavour sweet minty spearmint carvone caraway
**7**	s-Nicotine	54-11-5	B, A	Nicotine
**8**	Vanillin	121-33-5	B, A	Flavour Vanilla, vanillin, sweet, creamy, spicy, phenolic and milky
**9**	beta-Nicotyrine	487-19-4	B	Nicotine impurity

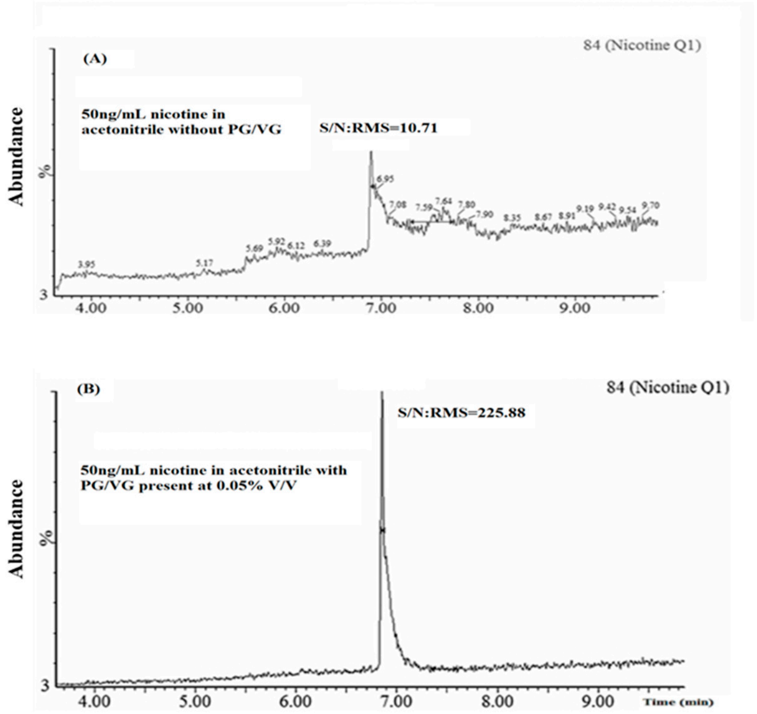

Supplementary Figure S1: Nicotine matrix effects (A) pure nicotine standard in acetonitrile (B) pure nicotine standard in acetonitrile with PG/VG present

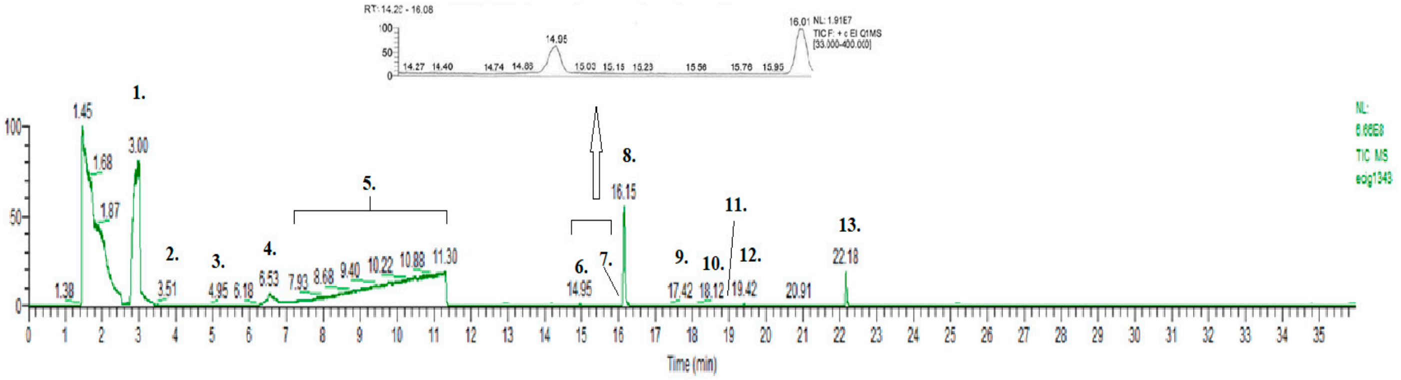

Peak	Name	CAS	Notes
**1**	1,2-PROPANEDIOL	57-55-6	Diluent propylene glycol
**2**	Ethyl butyrate	105-54-4	Flavour: Fruity, sweet, tutti frutti, apple, fresh and lifting, ethereal
**3**	Ethyl isovalerate	108-64-5	Flavour: Sweet, fruity, spice, metallic and green with a pineapple and apple lift
**4**	Maltol	118-71-8	Flavour: Sweet, cotton candy, caramellic, with jammy fruity and berry notes
**5**	Glycerin	56-81-5	Diluent glycerol
**6**	Butanoic acid, 2,3-dihydroxypropyl ester	557-25-5	unknown
**7**	Methyl anthranilate	134-20-3	Flavour: Sweet, fruity, concord grape, with a musty and berry nuance
**8**	s-nicotine	54-11-5	S-nicotine
**9**	Vanillin	121-33-5	flavour-vanilin
**10**	Dimethyl anthranilate	85-91-6	Flavour: Fruity grape skin, anthranilate-like with a woody and floral nuance
**11**	gamma-decalactone	706-14-9	Flavour: fruity creamy, peach flavour-gamma decalactone
**12**	PYRIDINE, 3-(1-METHYL-1H-PYRROL-2-YL)-	487-19-4	Minor alkaloid beta-nicotyrine
**13**	WS-3 (Cyclohexanecarboxamide, N-ethyl-5-methyl-2-(1-methylethyl)-)	39711-79-0	Flavour: Intense lingering cooling trigeminal effect. The cooling sensation slowly but steadily grows to a lingering cooling mouth feel with a slightly camphoraceous and minty character

Supplementary Figure S2: Fruit sample (“Frozen Grape”) chromatogram generated through non targeted analysis prior to degradation.
